# Supplementary material for: Fatty acid synthase inhibition alleviates lung fibrosis via β-catenin signal in fibroblasts
Source: Life Sci Alliance. 2024 Nov 20;8(2):e202402805. doi: 10.26508/lsa.202402805 (PMC11579593; doi:10.26508/lsa.202402805)

Fig.1C

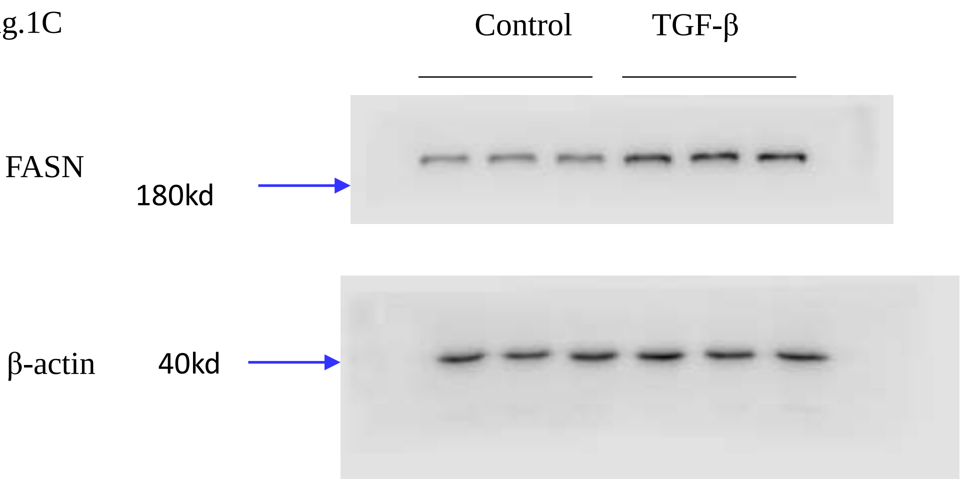
Control
TGF-β
FASN
180kd
β-actin
40kd

Fig.1F

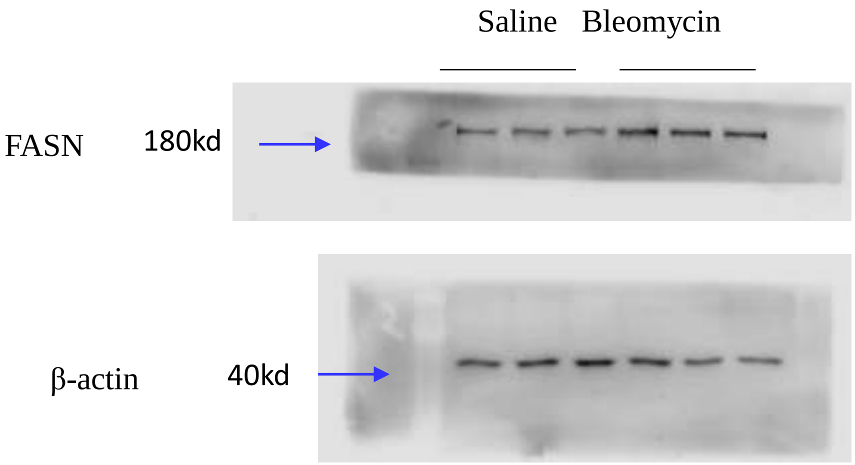
Saline
Bleomycin
FASN
180kd
β-actin
40kd

Fig.2E

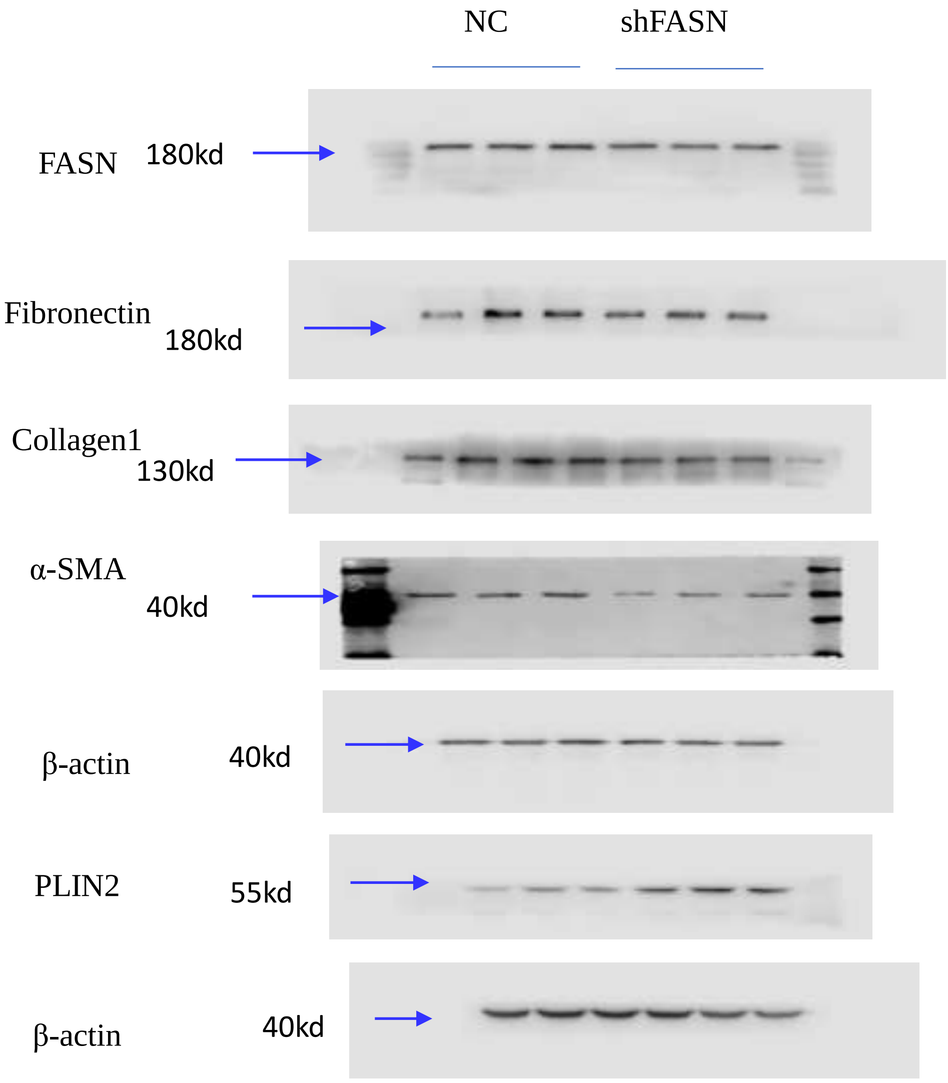
NC
shFASN
FASN
180kd
Fibronectin
180kd
Collagen1
130kd
α-SMA
40kd
β-actin
40kd
PLIN2
55kd
β-actin
40kd

Fig.2L

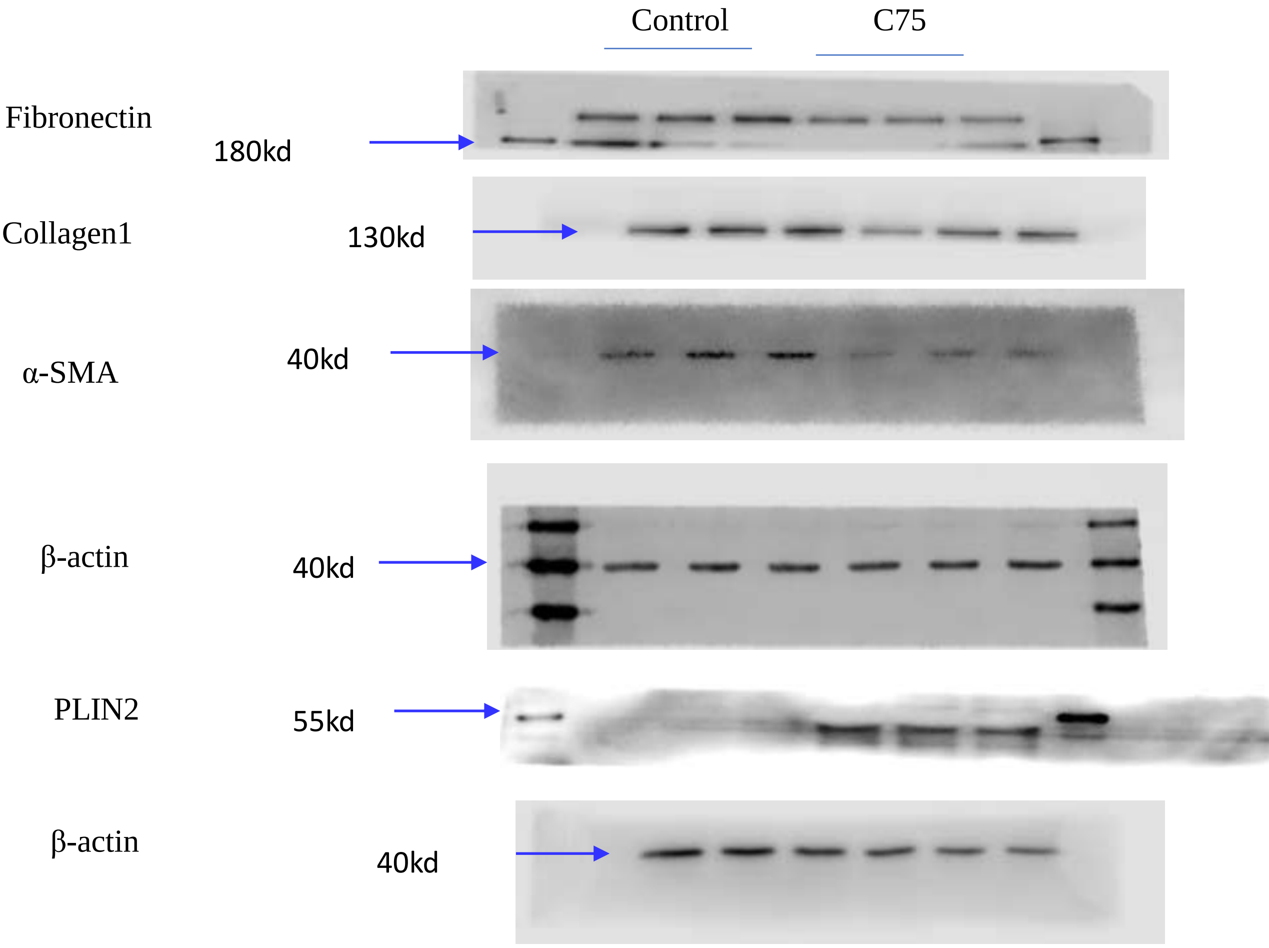
Control
C75
Fibronectin
180kd
Collagen1
130kd
α-SMA
40kd
β-actin
40kd
PLIN2
55kd
β-actin
40kd

Fig.3B

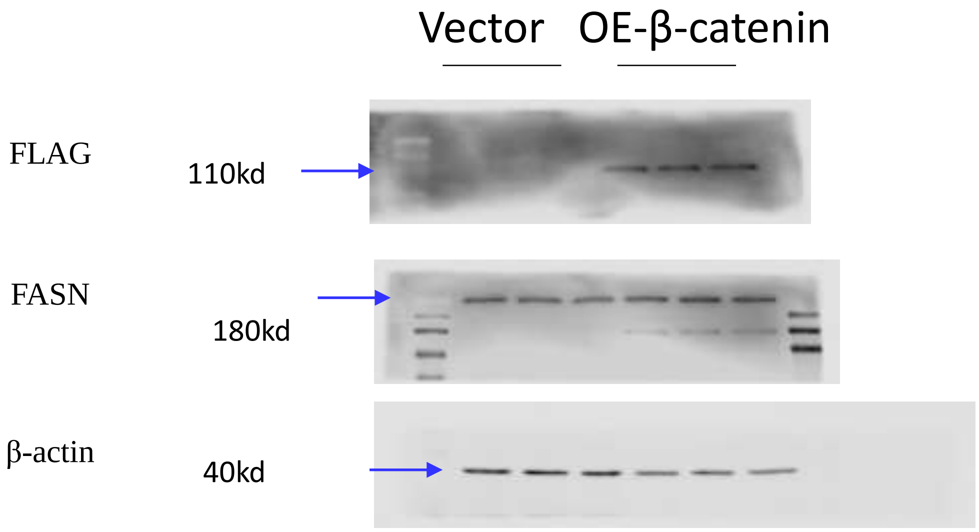

Fig.3E

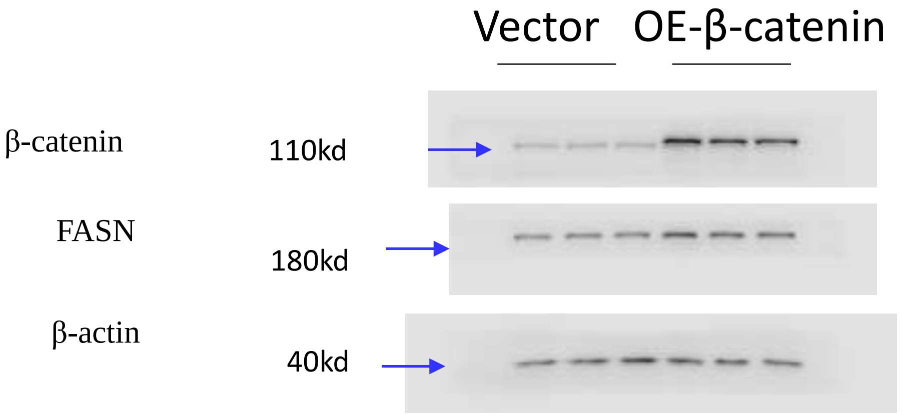

Fig.3H

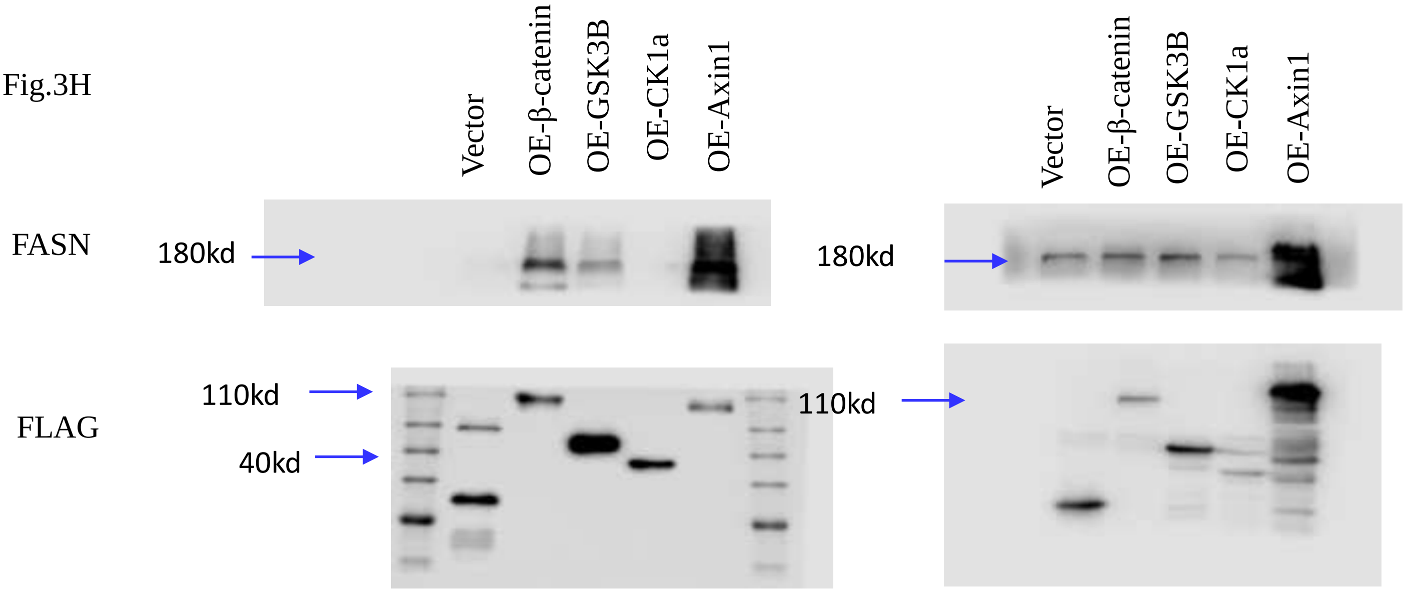

Fig.4B

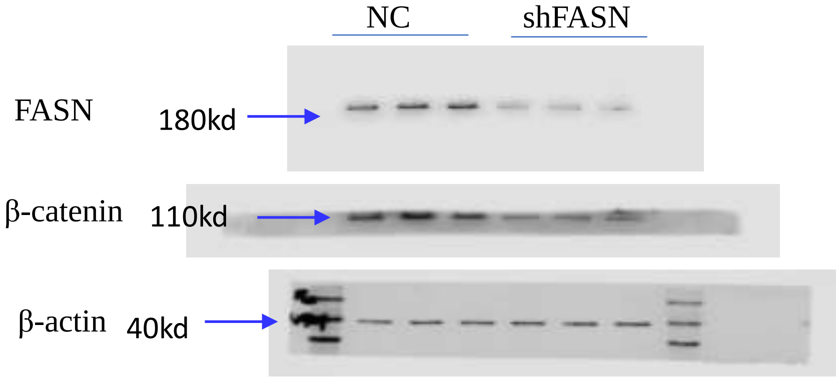

Fig.4D

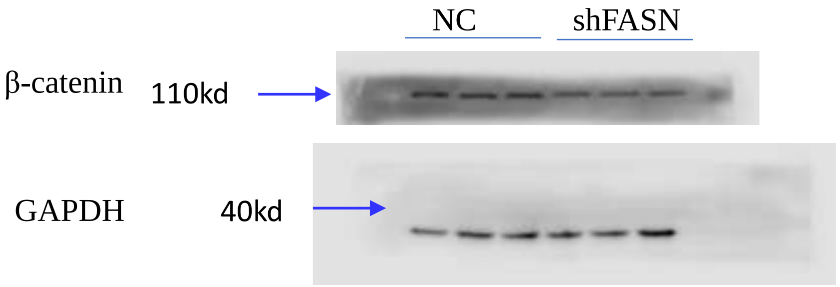

Fig.4E

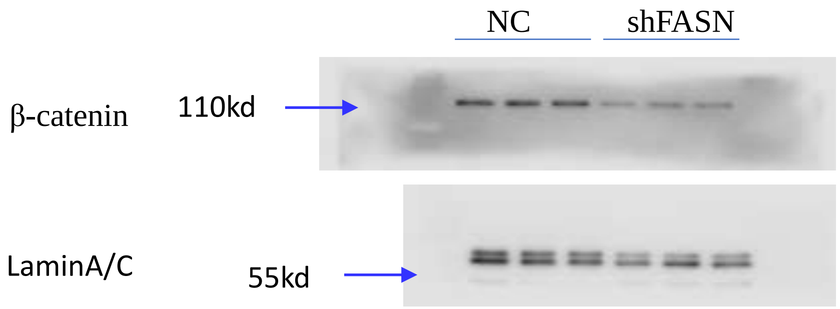

Fig.4H

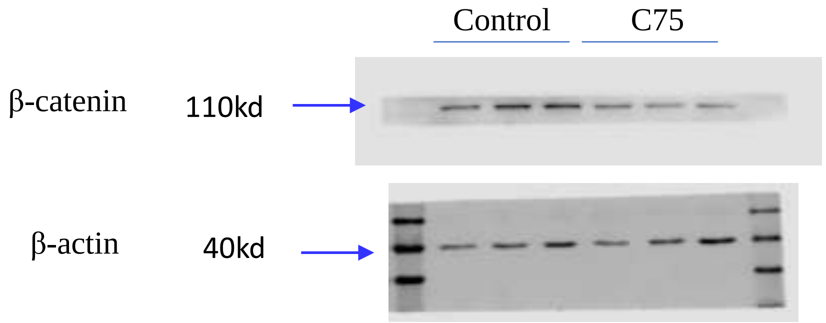

Fig.4J

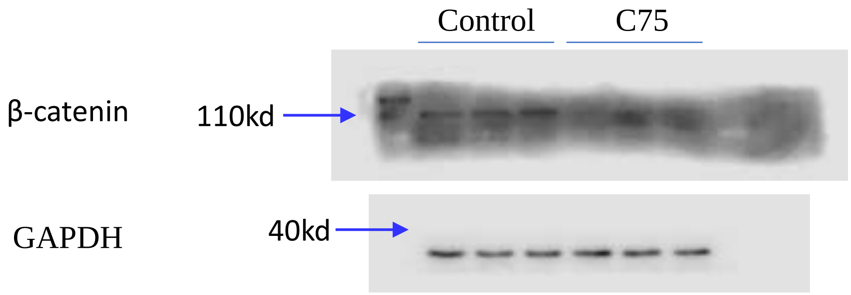

Fig.4K

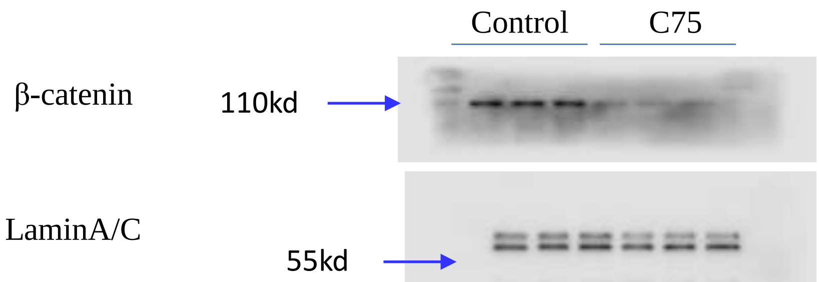

Fig.5C

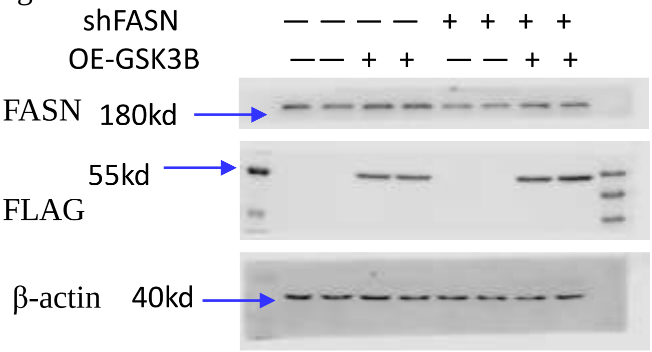

Fig.5D

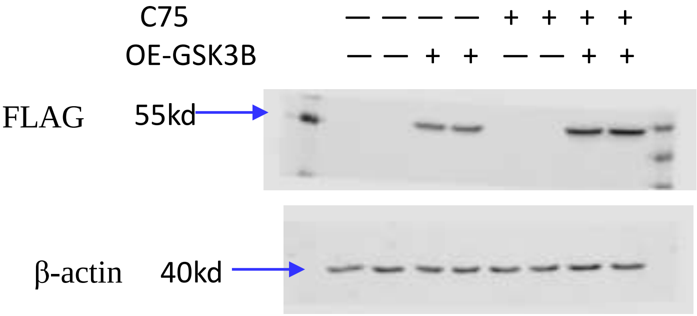

Fig.5E

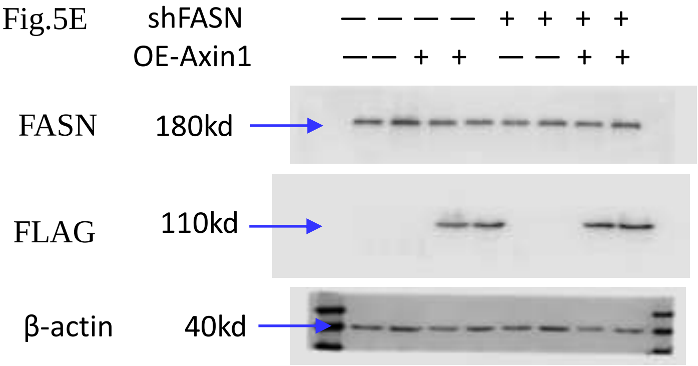

Fig.5F

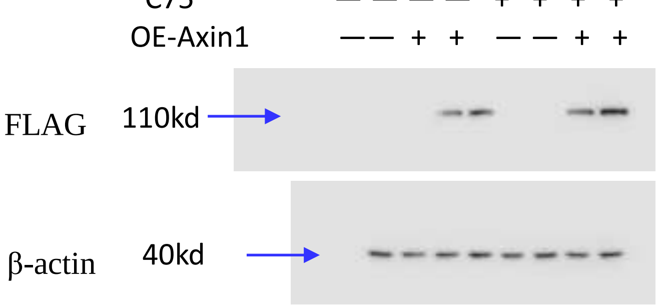

Fig.5I

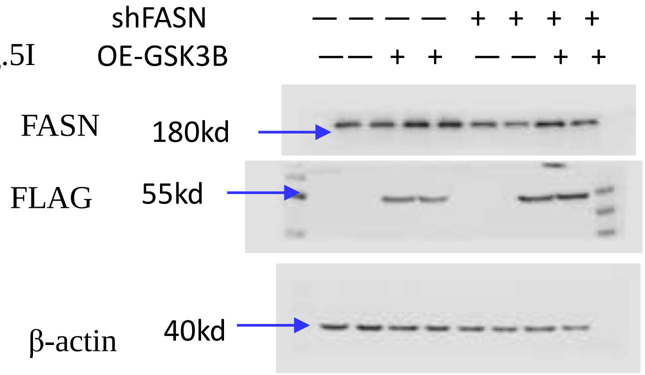

Fig.5J

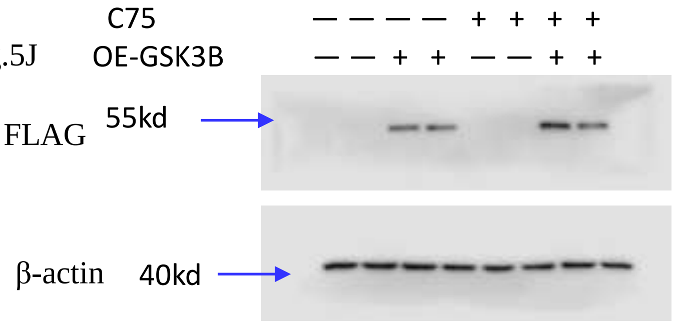

Fig.5K

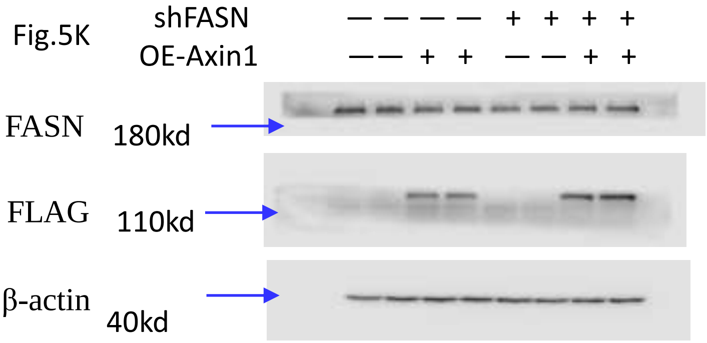

Fig.5L

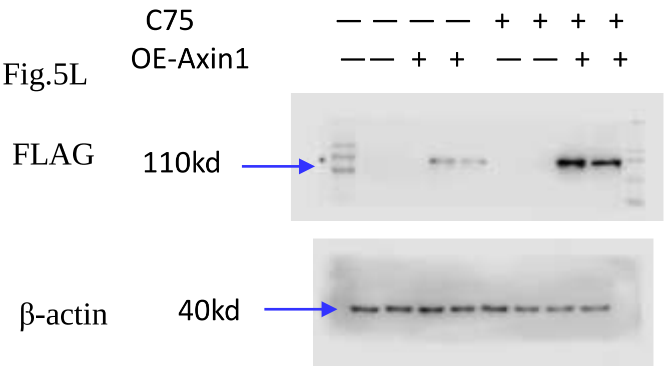

Fig.6D

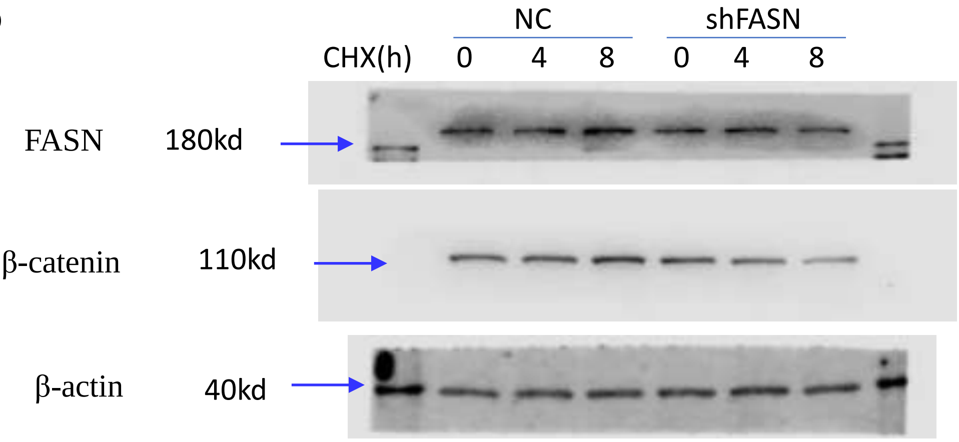

Fig.6E

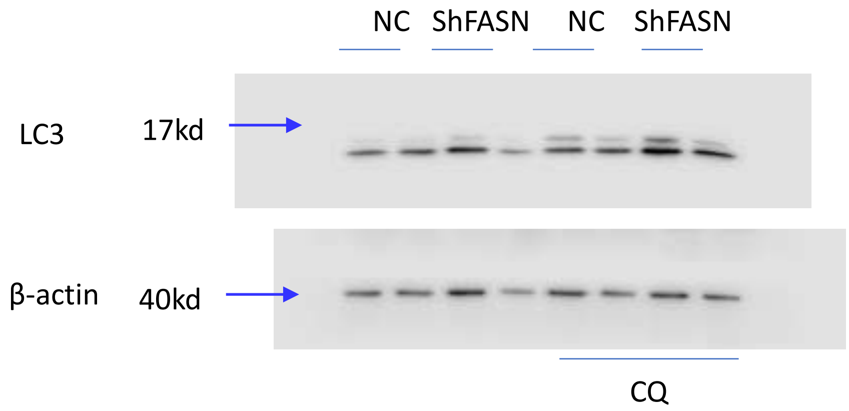

Fig.6J

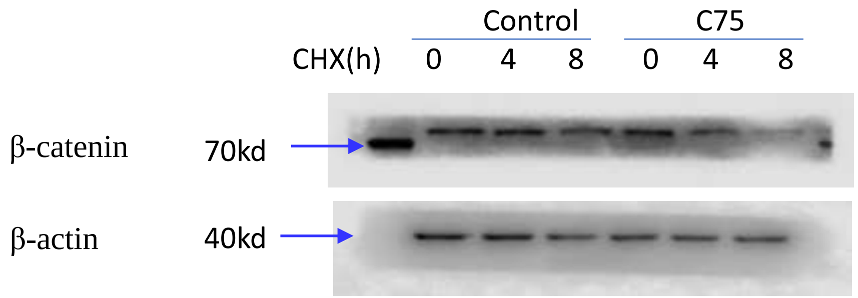

Fig.6K

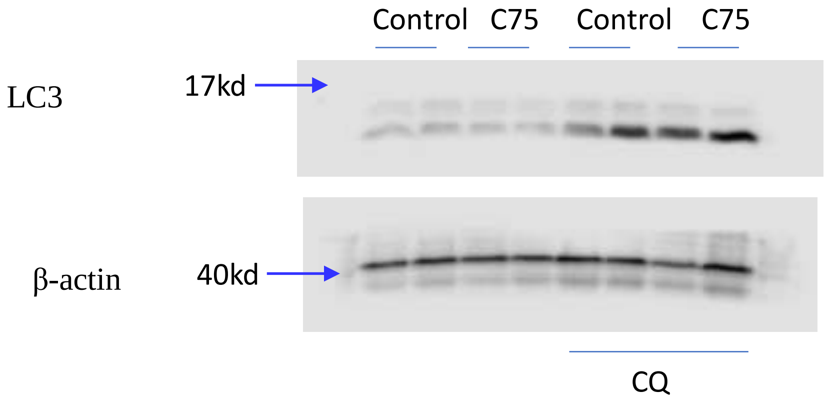

Fig.7H

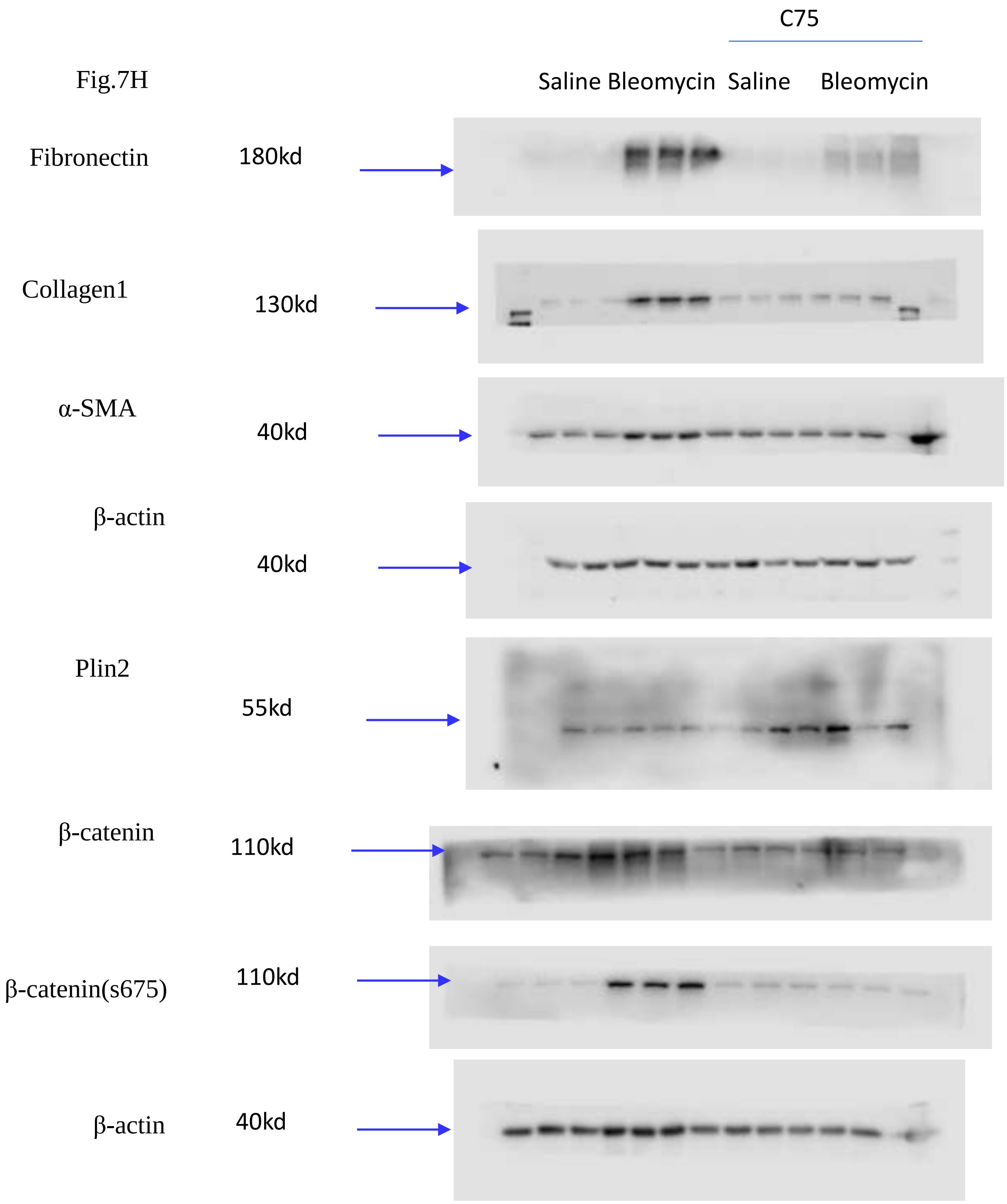
C75
Saline Bleomycin Saline Bleomycin
Fibronectin 180kd
Collagen1 130kd
α-SMA 40kd
β-actin 40kd
Plin2 55kd
β-catenin 110kd
β-catenin(s675) 110kd
β-actin 40kd

Suppliment Fig.1A

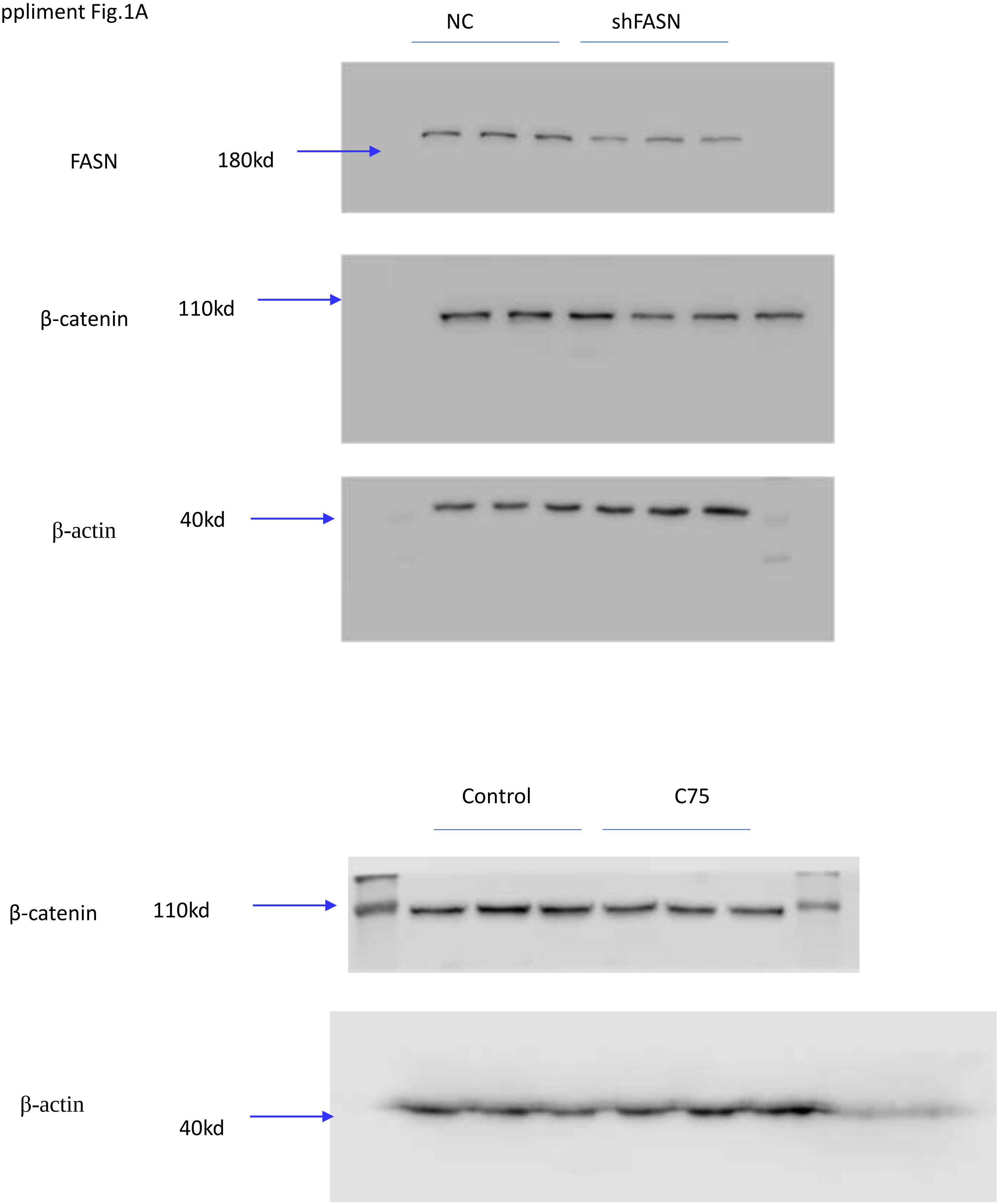

Supplement: Supplementary file 1 [file LSA-2024-02805_SdataF1_F2_F3_F4_F5_F6_F7_FS1.pdf]
